# The Role of Psychotherapy in the Care of Patients with Myalgic Encephalomyelitis/Chronic Fatigue Syndrome

**DOI:** 10.3390/medicina59040719

**Published:** 2023-04-06

**Authors:** Tilman Grande, Bettina Grande, Patrick Gerner, Sabine Hammer, Michael Stingl, Mark Vink, Brian M. Hughes

**Affiliations:** 1 Independent Researcher, 69117 Heidelberg, Germany; bettina.grande@gmx.de; 2 Klinik für Kinder und Jugendmedizin, Ortenau Klinikum, 77654 Offenburg, Germany; patrick.gerner@ortenau-klinikum.de; 3 Gesundheit & Soziales, Hochschule Fresenius, 65510 Idstein, Germany; sabine.hammer@hs-fresenius.de; 4 Facharztzentrum Votivpark, 1090 Wien, Austria; ordination@neurostingl.at; 5 Independent Researcher, 1096 HZ Amsterdam, The Netherlands; markvink.md@outlook.com; 6 School of Psychology, University of Galway, H91 TK33 Galway, Ireland; brian.hughes@universityofgalway.ie

**Keywords:** chronic fatigue syndrome, cognitive behavioral therapy, exercise, myalgic encephalomyelitis, pacing, psychotherapy, post-exertional malaise

## Abstract

Myalgic encephalomyelitis/chronic fatigue (ME/CFS) is a post-infectious, chronic disease that can lead to severe impairment and, even, total disability. Although the disease has been known for a long time, and has been coded in the ICD since 1969 (G93.3), medical research has not yet been able to reach a consensus regarding its physiological basis and how best to treat it. Against the background of these shortcomings, psychosomatic disease models have been developed and psychotherapeutic treatments have been derived from them, but their empirical testing has led to sobering results. According to the current state of research, psychotherapy and psychosomatic rehabilitation have no curative effect in the treatment of ME/CFS. Nevertheless, we see numerous patients in practices and outpatient clinics who suffer severely as a result of their illness and whose mental well-being and coping strategies would benefit from psychotherapeutic help. In this article, we outline a psychotherapeutic approach that serves this need, taking into account two basic characteristics of ME/CFS: firstly, the fact that ME/CFS is a physical illness and that curative treatment must therefore be physical; and secondly, the fact that post exertional malaise (PEM) is a cardinal symptom of ME/CFS and thus warrants tailored psychotherapeutic attention.

## 1. Introduction

ME/CFS has been a little-noticed disease for many years, but has attracted increased attention during the pandemic because it has many symptomatic overlaps with the clinical representation of long COVID [1,2]. It is estimated that up to 50% of long COVID patients may have the clinical presentation of ME/CFS [3]. Affected individuals present with a variety of symptoms, including feelings of fatigue, lack of strength, muscular pain, headaches, sleep disturbances, non-restorative sleep, hyper sensitivity to sensory stimuli of all kinds, digestive problems, as well as cognitive symptoms, such as impaired concentration or memory and a feeling of fogginess, which is vividly described by the term ‘brain fog’. A key feature is post-exertional malaise (PEM), which refers to the delayed decline in health after physical, mental, or psychological demands—of this, we will return to in greater detail.

There are various options for the medical treatment of ME/CFS, but their indications and effectiveness are insufficiently tested [3,4]. There are no standards for the treatment of ME/CFS patients. In the absence of somatic treatment approaches, models for understanding ME/CFS and therapeutic approaches based on them were developed by clinical psychologists as early as the 1990s [5,6]. These approaches are based on the assumption that ME/CFS sufferers have dysfunctional cognitions: they are overly focused in their attention on physical discomfort, which they attribute to their disease and anxiously anticipate as a consequence of effort. They, therefore, avoid activities and thereby circularly reinforce their fears. The therapeutic approach based on this, therefore, aims—on the one hand—to modify these problematic cognitions (cognitive behavior therapy, i.e., CBT) and—on the other hand—to eliminate avoidance through gradual activation (graded exercise therapy, i.e., GET). This approach has been evaluated in an extensive study [7]; however, this only showed minor effects, which have been qualified as questionable under a method-critical view [8,9]. It must be added that the question of the psychological or psychosomatic backgrounds of ME/CFS is discussed very emotionally, because those affected vehemently resist a ‘psychologisation’ of their disease. This is based on the worry of being pushed into a psychosomatic category by a helpless somatic medicine and being forgotten there [10]. In addition, there are repeated reports of patients who have experienced a worsening of their complaints under GET.

In October 2021, the British health authority, NICE (the National Institute for Health and Care Excellence), published an updated guideline recommending that activation therapy (GET) should not be used in the psychosomatic rehabilitation for ME/CFS-patients, on the grounds that it risks a deterioration of the disease state in affected individuals. Furthermore, NICE proposed that CBT be only offered to ME/CFS patients as a supportive measure, on the basis that CBT itself does not produce any curative effect in ME/CFS [11]. In Germany, the responsible Institute for Quality and Efficiency in Health Care (Institut für Qualität und Wirtschaftlichkeit im Gesundheitswesen, i.e., IQWiG) inferred a weak effect for both GET and CBT in a first draft based on very few studies [12]. However, careful examination of these studies and the arguments put forward by IQWiG suggest that this assessment and the recommendations (already very cautiously worded by the IQWIG itself) are very problematic because they ignore IQWiG’s own methodological handbook, as well as the absence of evidence for objective post-GET/CBT improvement in the examined studies [13,14].

Given these facts, it seems reasonable to conclude that psychotherapy cannot play a curative role in ME/CFS patient care. This not only applies to the two methods mentioned above, but also to other well-established forms of psychotherapy that share central conceptual elements of GET and CBT, and which are critical or even harmful to ME/CFS patients—a point that we will come back to later. However, despite this, we see an increasingly large number of ME/CFS patients in the practices, and in outpatient clinics who are suffering from intense psychological distress and are in urgent need of psychological support. These patients seek our help either because they have developed comorbid psychological symptoms, because they have fallen ill while undergoing psychotherapy, or because their disease has been misdiagnosed as ‘psychosomatic’ by medical practitioners, and thus they have subsequently been referred to psychotherapy. Against this backdrop, we outline below an approach to psychotherapy for ME/CFS that can provide affected individuals with valuable assistance and which avoids the problematic alignment of the standard treatment approaches for ME/CFS. Psychotherapy of this kind requires a fundamental change in perspective, necessitating a more appropriate therapeutic attitude to the specific characteristics of ME/CFS, which we will now explain in more detail.

## 2. The Psychotherapeutic Attitude When Working with ME/CFS Patients

ME/CFS-adapted psychotherapy is founded on the understanding that ME/CFS is an unambiguously somatic disease [11,15] and that any ultimately effective treatment must be a somatic one. Therefore, core elements of any psychotherapeutic work must be focused on teaching the specific technique of pacing as the effective method for managing symptoms and health setbacks during the course of the disease. Appropriate psychotherapy will also focus on managing the impact of the disease on the lives of those affected. This fundamental comprehension that ME/CFS is a physical disease is crucial because it delimits both patients’ and therapists’ expectations regarding treatment, and thus establishes a framework within which psychotherapeutic work can be possible as a standard that is appropriate for tackling emotional needs and the support of pacing. By now, there is an vast body of empirical findings from many areas of somatic medicine that demonstrate the physiologic nature of ME/CFS [1,2,3,4,11,12,13,14,15]. Nonetheless, there is still no clear single diagnostic biomarker for the disease and no scientific consensus on the underlying mechanisms of pathology. Additionally, currently, proponents of psychotherapy or psychosomatic rehabilitation as ‘curers’ for ME/CFS use this lack of clarity as an opportunity to privilege their own therapies [16,17]. In doing so, untested claims about the possibilities of psychotherapeutic-psychosomatic treatment are commonplace. This is perverse because extraordinary restraint is necessary when dealing with ME/CFS given that affected persons are otherwise expected to do (and promised they will achieve) something that they cannot plausibly ever do given their physical limitations.

Recognizing and gaining a firm understanding of post-exertional malaise (PEM) as a cardinal symptom of ME/CFS is also pivotal [11,14,15,18]. PEM refers to a symptomatic deterioration after minimal physical, cognitive, or emotional exertion, and which forces individuals into complete passivity and occurs immediately, or with a time delay of up to 48 h. However, this is not in any way the type or level of fatigue seen in other illnesses, such as in depression or in patients during or after chemotherapy. In contrast, PEM in ME/CFS is a severe health breakdown (or ‘crash’) with diverse symptoms, such as a flu-like feeling of illness, muscle pains, headaches, extreme sensitivity to stimuli of any kind, sleep disturbances, and cognitive symptoms (e.g., ‘brain fog’). Recovery from a crash such as this can take days, weeks, months, or more. Moreover, each crash is accompanied by the uncertainty of whether the previous state can ever be regained or whether the deteriorated condition is permanent.

As such, crashes must be avoided, and psychological support should aim to assist patients in doing so. Pacing is one such activity management strategy by which to help ME/CFS patients, first described by health psychologist Goudsmit in 1989 [19]. The primary goal of pacing is to foster the ability or skill to not exceed the individual’s given limits. Pacing also provides stability to sufferers as it helps manage their symptomatology [20]. Unfortunately, PEM is not easy to understand for sufferers and practitioners alike because, contrary to all common understanding, a seemingly vigorous state of well-being does not guarantee that an activity undertaken based on this state of well-being will not lead to a sudden and severe decline in health. Repeatedly, this leads sufferers to overtax themselves spontaneously and encourages practitioners to give advice that, in time, turns out to be harmful.

## 3. Key Psychotherapeutic Elements of Working with ME/CFS Sufferers

Psychotherapeutic work with individuals suffering from ME/CFS will ideally involve a complex and individually tailored learning approach to pacing. This fundamental approach monitors and limits the different types of exertion and critical activities that can trigger symptoms—whether they are physical, mental, or emotional, and whether they are enjoyable activities or negative stressors. Such triggering activities can vary significantly from person to person [18,19]. This is especially important because it is not always clear what a patient’s limit exactly is, even more so because it can vary from day to day. Once pacing has been successfully established as a skill, affected persons can prevent deterioration and thus use the scope available within their limits in a less dangerous way. Therapists can accompany and promote this process, but they do not primarily have superior knowledge that would allow them to guide the affected person ‘from the outside’, as it were, and to, for example, encourage them to increase their activity. Even patients gifted with self-awareness or body awareness may need a long time until they have developed disease management in the sense of pacing that suits them, and is beneficial. This underlines the importance of restraint and patience in working with these patients.

Pacing requires ME/CFS patients to exercise a high degree of vigilance and personal discipline, often needing to give up a large part of their daily routines. As the disease progresses, patients must often adjust or abandon their expectations, projects, and, sometimes, even their life plans due to their uncertain future outlook. Understandably, many patients react to these changes with impatience, internal resistance, and despair as their illness progresses [21,22]. In addition, patients often feel subjective or objective pressures from their social environment (such as from family, friends, work, insurance, and even medical professionals) to return to ‘normality’, a pressure that increases as the illness persists [10]. As a result, the key points of psychotherapeutic work with ME/CFS patients should include the following:

- The introduction and practice of the principle of pacing, i.e., the perception of individually significant stress situations and their delayed consequences in the form of crashes, the anticipation of these consequences, and the control of the relevant current behavior;
- Dealing with the inner resistance to the often severe constraints that pacing demands of each individual and the need for varying degrees of self-discipline;
- Addressing negative attributions and reactions to pacing in the social environment, which may threaten social resources, as well as identifying and utilizing available resources for self-care and setting boundaries against social pressure;
- Existential themes related to being affected by a severe, chronic, and potentially debilitating illness, as well as therapeutic support for and coping with the associated suffering.

We believe that experienced psychotherapists will find themselves back in familiar territory when dealing with these themes and will have many clinical techniques at their disposal to address them. However, there are further distinctive characteristics relating to ME/CFS that are unfamiliar and require some more detailed explanations. These include issues relating to the (un)certainty of diagnostics, cooperation with physicians, personality-specific challenges in conveying and practicing pacing. A special challenge is the adaptation of the general conditions of psychotherapeutic practice concerning the limits of severely impaired patients. This concerns the manner of scheduling appointments, the duration and frequency of sessions—face to face vs. video/telephone—and the content and gravity of the issues addressed, which all need to be adapted to the (stress) limits of these patients. However, going into these details is beyond the scope of this brief overview.

Therefore, what can be achieved with the help of pacing? To begin with, stability, which affected individuals appreciate very much because it allows them to prevent crashes and to improve their ability to manage their symptoms, giving them back a sense of control and self-confidence. In addition, patients can avoid further deterioration and achieve tangible improvement by making the most of their abilities within their individual constraints. It is important to realize that pacing is a way of trying to prevent symptom exacerbations and relapses. It is not a treatment that will lead to recovery or a significant improvement in function.

## 4. Discussion

To understand the approach presented, it is crucial to clarify the differences to the usual psychotherapeutic procedure. As previously stated, psychotherapists do not possess superior knowledge that would allow them to guide patients from an external perspective. Instead, the practice of pacing is wholly based on the perception and self-awareness of patients, who essentially become the leading experts regarding their illness and pacing. Thus, established interventions commonly used in the psychotherapy of somatoform disorders or in psychosomatic rehabilitation treatments are inadmissible.

Psychotherapeutic work with ME/CFS sufferers aims at recognizing and acknowledging (stress) limits. This contradicts the customary attitude of encouraging patients to extend and exceed their limits during treatment, which is widely applied across many psychotherapeutic methods. This is why, for example, the concept of GET is ill-suited for ME/CFS, as by presupposing the therapeutic benefit of activating measures and the gradual expansion of limits, it violates the fundamental principle of pacing: observing one’s limits with particular care and protecting those that are affected. This precaution should apply not only to physical activation as in the case of GET, but also to any other type of exertion, whether physical, psychological, or mental. In ME/CFS, each confronting element of psychotherapy is therefore potentially risky.

Moreover, systematically questioning a patient’s perceptions of stress limits in terms of so-called dysfunctional cognitions (a concept often invoked during CBT) or of unconscious motives (as usually focused in psychodynamic therapies) is contraindicated in ME/CFS. That kind of approach encourages individuals to distrust their perceptions of stress limits, in direct contradiction to the principle of paying close attention to, and respecting, such limits as encapsulated by the notion of pacing. It is especially problematic when psychotherapists encourage patients to misconstrue the phenomenon of PEM itself as the product of ‘dysfunctional’ cognition [23]. This too subverts the core principles of pacing by presenting the individual’s perception of the deferred consequences of effort as a cognitive error (a ‘mind thing’) that needs remedial action.

## 5. Conclusions

There are several important and clear distinctions to be made between the pacing-led ME/CFS treatment approach that is outlined above and in many of the so-called ‘standard’ psychotherapeutic techniques that are often used with patients in health contexts. Challenging patients’ cognitions and stress limits is so elementary to routine psychotherapeutic/psychosomatic care that doubts about so-called ‘modifications’ in the sense of a ‘particularly careful’ approach to ME/CFS patients are, in our opinion, justified. However, conventional approaches involve unfulfillable therapeutic promises and the serious danger of deterioration. We, therefore, believe that a radical paradigm shift is needed in psychotherapy, health psychology, and other fields where psychosocial and behavioral support is provided to ill people, and which recognizes the somatic nature of ME/CFS and adjusts therapeutic goals accordingly. A particular imposition of ME/CFS is the phenomenon of PEM, whose control by means of pacing demands a high degree of vigilance, discipline, and renunciation from the patients. This means an enormous challenge for those affected, which is not encountered in this specific way in other chronic diseases. A psychotherapy that takes these realities into account and offers help in coping with them can make an important and, in our view, an indispensable contribution to the care of ME/CFS patients.

## Data Availability

Not applicable.
